# A Global Airport-Based Risk Model for the Spread of Dengue Infection via the Air Transport Network

**DOI:** 10.1371/journal.pone.0072129

**Published:** 2013-08-29

**Authors:** Lauren Gardner, Sahotra Sarkar

**Affiliations:** 1 School of Civil and Environmental Engineering, University of New South Wales, Sydney, NSW, Australia; 2 National ICT Australia (NICTA), Sydney, NSW, Australia; 3 Section of Integrative Biology, University of Texas at Austin, Austin, Texas, United States of America; Centro de Pesquisas René Rachou, Brazil

## Abstract

The number of travel-acquired dengue infections has seen a consistent global rise over the past decade. An increased volume of international passenger air traffic originating from regions with endemic dengue has contributed to a rise in the number of dengue cases in both areas of endemicity and elsewhere. This paper reports results from a network-based risk assessment model which uses international passenger travel volumes, travel routes, travel distances, regional populations, and predictive species distribution models (for the two vector species, *Aedes aegypti* and *Aedes albopictus*) to quantify the relative risk posed by each airport in importing passengers with travel-acquired dengue infections. Two risk attributes are evaluated: (i) the risk posed by through traffic at each stopover airport and (ii) the risk posed by incoming travelers to each destination airport. The model results prioritize optimal locations (i.e., airports) for targeted dengue surveillance. The model is easily extendible to other vector-borne diseases.

## Introduction

Dengue is the most common mosquito-borne viral disease in the world [1]. According to the World Health Organization [2]: “In 2012, dengue ranked as the fastest spreading vector-borne viral disease with an epidemic potential in the world, registering a 30-fold increase in disease incidence over the past 50 years.” An increase in dengue occurrence in many of the endemic regions worldwide, in conjunction with a significant rise in the volume of international air travel, has resulted in an increased likelihood of imported dengue infections among travelers returning from dengue-endemic regions [3]. It has also increased the potential for transport and establishment of populations of the mosquito vector species in those regions in which they may not currently be present but suitable habitat is available.

The infectious agent for dengue is a virus that is transmitted between human individuals through the bite of infected Aedes mosquitoes (mainly *Ae*. *aegypti* and *Ae. albopictus*), with humans also serving as the main viral host [2]. The geographic establishment of dengue is believed to be limited by the occurrence of its principal vector mosquito species, *Ae. aegypti* and *Ae. Albopictus* [4]. Both species have proven to be highly adaptable to human habitation and, as a result, the global spread of the vector has been difficult to contain [2]. Dengue is considered endemic to urban and suburban areas in parts of tropical and subtropical America, part of Australia, South and Southeast Asia, the Pacific, and eastern Africa. In addition, the number of imported cases of dengue in the U.S. and Europe is on the rise and further spread and establishment is anticipated [3]–[5].

At present, there is little epidemiological surveillance for dengue anywhere outside endemic areas, for instance, none on a national scale in Europe or at the state level in the United States [5]. Limiting the importation and establishment of dengue in such new areas will require dedicated surveillance measures, ideally based on reliable models of vector presence and virus incidence. This paper presents a model which quantifies the relative risk of dengue importation and establishment posed by each airport based on the likely presence of dengue-infected travelers. The model incorporates the arrival of potentially infected travelers at both stopover and destination airports along travel routes originating in dengue-endemic regions. The results from the model can be used to prioritize airports at which dengue surveillance efforts should be directed. Our network-level model uses international passenger travel volumes, travel routes, travel distances, regional populations, and predictive species distribution models (for the two vector species) to quantify the risk posed by both stopover and destination airports. All global origin-destination (OD) travel pairs are evaluated and all major world airports are considered. The analysis also provides guidance for the type of data collection efforts that must be undertaken to enhance the predictive accuracy of such models. The model can also be used to evaluate the effects of changes in passenger travel routes and volumes on spatial patterns of infection spread.

The model compounds all modes of dengue infection which can be caused by four virus serotypes (DENV-1, DENV-2, DENV-3, and DENV-4), and can range in clinical manifestation from asymptomatic infection to severe systemic disease [2]. Dengue fever (DF) is the more common manifestation of the disease (with an estimated 50 million infections occurring annually world-wide in over 100 countries, more than malaria [2]), while dengue haemorrhagic fever (DHF) and dengue shock syndrome (DSS) are rarer and much more severe manifestations. The model presented in this paper does not distinguish between DF, DHF, and DHS cases because the available data do not permit a more fine-grained analysis.

Even in the parts of the world where dengue is now rare, e.g., the United States and Europe, the mosquito vectors are still present. For example, at least one of the two major vector species, *Ae. aegypti* or *Ae. albopictus*, is known to have established populations in 26 U.S. states [6]. The European Center for Disease Control [7] recently gathered entomological and environmental data to map the current distribution, as well as establishment risk, for *Ae. albopictus* in Europe in the event of its introduction. It concluded that temperate strains of this species already exist and are likely to spread with new populations becoming established in several parts of Europe [7]. Further, the International Air Travel Association [8] forecasts that international passenger numbers will grow from 1.11 billion in 2011 to 1.45 billion in 2016, with the dengue-endemic regions of Latin America, Africa and the Asia-Pacific region representing three of the top five fastest growing regions.

Thus, imported cases of dengue via international travel may potentially result in establishment of an autochthonous disease cycle and new regional outbreaks in previously non-endemic regions. This can occur in at least two ways: (i) locally established mosquito populations become infected from new hosts (infected travelers) and then spread the disease; or (ii) mosquitoes carrying the virus arrive at a new environment suitable for them. This threat was exemplified recently in Key West, Florida, which experienced sizeable local outbreaks of autochthonous dengue transmission in 2009–2010 [9]. There have also been dengue outbreaks in south Texas, along the Texas-Tamaulipas border, but air travel is unlikely to have had a role in these outbreaks [10]. Additionally, in late 2012, Europe suffered its first sustained outbreak since the 1920s, with 2,000 people infected on the Portuguese Atlantic island of Madeira [2].

Further complications arise from the severe underestimation of dengue cases due to under-reporting and passive surveillance in both endemic and non-endemic regions. In tropical and subtropical countries where dengue fever is endemic, under-reporting may be due to misdiagnosis, limitations of the standard World Health Organization (WHO) case classification, and lack of laboratory infrastructure and resources, among other factors [11]. In non-endemic regions such as the U.S. and Europe, the actual number of dengue infections is greatly underestimated due to unfamiliarity with the disease. Additionally, 40–80% of all dengue infections are asymptomatic or closely mimic flu symptoms for which they are mistaken.

This lack of accurate infection data makes it difficult to assess the actual threat of the disease. Epidemics of dengue, their seasonality, and oscillations over time, are reflected by the epidemiology of dengue in travelers [3]. Modern transportation bridges the natural barriers previously responsible for containing infected vectors to specific geographic regions. For example, the global movement of troops and cargo ships during World War II facilitated the dissemination of Aedes mosquitoes and resulted in substantial spread of the disease in Southeast Asia [12]. Transportation of used tires has been held responsible for introducing *Ae. albopictus* into the U.S. from Brazil in the 1980s [3].

Various studies have been conducted to identify the highest travel risks. One survey conducted by the European Network on Imported Infectious Disease Surveillance program, analyzed 294 patients with DF for epidemiological information and clinical features from January 1999 through December 2001 [13]. They found most infections were imported from Asia. Tatem *et al*. [14] estimated the relative risk of the importation and establishment of *Ae. albopictus* by sea and air routes, based on normalized measures of traffic and climatic similarity, and found a strong positive correlation between the historic spread of *Ae. albopictus* (into new regions) and a high volume of shipping (routed from ports where the species was already established). The total volume of travel was determined by the number of ship visits for sea travel and passenger volume for air travel. The climatic similarity was calculated as a distance-based vector. While their work provided insight into the vector importation and establishment process, model validation remained qualitative. Moreover, Tatem *et al*.’s approach addressed the risk of importation and establishment of vector species but not the likelihood of infection directly.

Gardner et al. [4] extended the work by Tatem and collaborators by complementing qualitative risk analysis with quantitative model calibration using infection data, thus taking infected individuals into account. Gardner et al. incorporated climatic factors using species distribution models which are more robust than statistical correlational analysis as relied upon by Tatem and collaborators [15]. This methodology has become standard in disease ecology and epidemiology [16]–[19]. The analysis that follows expands on the paper by Gardner et al. [4] in five ways:

- The model identifies high risk *airports*, whereas the previous model identified high risk *travel routes*. Quantifying the risk posed to travel routes and airports requires different modeling approaches. Furthermore different types of surveillance efforts and control policies are required to monitor each. The shift of emphasis to airports from travel routes is motivated by the assumption that monitoring and surveillance of airports would be easier to implement than travel routes.
- The model is able to differentiate between two different types of risk, destination risk and stopover risk. Each of these risk types will require different airport monitoring measures. High destination risk airports will require monitoring which identifies travelers exiting an airport, while high stopover risk will require monitoring travelers within an airport (between flights).
- The new model is more *spatially disaggregated*. Travel between each airport is modeled, rather than aggregating travel volumes across states or countries.
- The new model is *temporally disaggregated* from an annual to a monthly time scale. This allows the model to account for the impact of seasonality on travel risk. For reasons indicated below, we use June, 2011 in our analysis.
- In this paper all *global* airports are modeled, whereas the previous study was limited to destinations in the United States and the EU. This turned out to be a significant extension because a majority of the highest risk airports were identified as being located outside of the EU and the U.S.

More recently Nicolaides et al. [20] evaluated the risk posed to US airports using a stochastic agent-tracking model which detailed air traffic and the correlated nature of mobility patterns and waiting-time distributions of individual agents. The objective was to provide an accurate measure of the early-time spreading power of individual nodes, i.e. airports. However, the model only considered contact-based diseases and, therefore, did not incorporate any climactic or other ecological data. In this paper we seek to identify the set airports in which infected passengers are most likely to be present. This is accomplished by separately computing the risk posed by each stopover airport as a function of the passenger through traffic, and the risk posed by each destination airport as a function of the incoming passenger traffic. While Nicolaides et al. relied on Monte Carlo simulation to evaluate the risk, this paper uses analytical expressions to quantify the airport risk. Finally, while our analysis quantifies the relative risk of dengue infected (air travel) passengers arriving at airports, it does not include the importation of infected vectors since the influence of that possibility is yet to be established [2]–[3], [7].

## Methods

This section further illuminates the problem and introduces our species distribution model and network-based model for the risk analysis.

### 2.1 Data

The network model required four types of types of input data which are listed below. The species distribution models required data on the geographical occurrence of *Ae. aegypti* and *Ae. albopictus* and a suite of predictive environmental variables; these will be discussed in Section 2.2. The required data for the network model were:

1. Transportation data
2. City population data
3. The set of dengue-endemic regions
4. Output from the Species Distribution Models

The transportation data consisted of all global airports travel routes for each month. The spatial data consisted of travel distances for each travel route. The transportation and spatial data was obtained from the International Air Transport Association (IATA) [8], and included origin, destination and stopover airports for all routes, as well as the flight distances and calibrated passenger travel volumes for each route. The route-specific passenger travel volumes supplied by IATA were calibrated based on data from 240 airlines comprising 84% of global air traffic, and includes over 9000 airports [8]. The passenger volumes were available at a monthly temporal resolution, which thus determined the temporal resolution of the model. (In cases where travel data are available at a finer (i.e., more disaggregated) temporal resolution, the model can be used for the relevant (e.g., daily or weekly) time period.) The transportation data used in this paper were limited to passenger travel volumes and did not include cargo flights on which vectors could potentially be transported because the latter mode of dengue spread was excluded from this model. The analysis was conducted using June, 2011 travel data [8]. June was chosen based on the results of Tatem [21] who find it to be the month where climatically sensitive organisms moving on the worldwide airline network (WAN) will be most likely to find their arrival destinations habitable [21]. This climatic similarity during June appears to compensate for the fact that June is not at the height of the dengue season in many countries in the Southern hemisphere.

The corresponding populations for all cities serviced by a given airport were collected from multiple sources. For U.S Airports 2069 of the 2343 airports were assigned city populations based on the city in which the airport was located by using U.S. Census Data [22]. The remaining U.S. airports and 4147 of the 7353 non-U.S. world airports were matched by name using the Spatial Analyst extension of ArcMap10, and the WorldAirports.dat [23] and WorldCities.dat [24] databases. The remaining 3206 world airports that could not be matched by name were assigned populations of the closest city with a population greater than 15,000 using the Spatial Analyst extension and the Cities15000.dat [25] and WorldAirports.dat [23]. The data set including only cities with populations greater than 15,000 was chosen to capture the risk posed to the nearest medium- or large-sized town or city in the region served by the airport. Often, airports which serve major cities are located outside of the city center, thus if all named towns were considered, the model would incorrectly compute and attribute risk to a small town closer to the airport rather than the major metropolitan region actually served by the airport.

The set of dengue-endemic regions were based on those identified by the U.S. Center for Diseases Control (CDC) [26].

### 2.2 Species Distribution Models

The risk for the establishment of dengue and potential cases of disease in an originally non-endemic area depends fundamentally on the ability of a vector to establish itself in that area, that is, on the ecological conditions for the vector there. When these ecological conditions are suitable, the disease can become endemic in two ways: (i) if the vector is already established, it can become infected from a person infected with dengue arriving in that area; or (ii) infected vectors can be transported into such an area and establish themselves. For this process, habitat in that area must be ecologically suitable for that vector. A quantitative relative measure of the suitability of one area compared to another defines the relative ecological risk of that area [16]–[19]. If the ecological risk is low, such an establishment is highly unlikely. If that risk is high, then other factors, such as the (temporally) immediate ambient environmental conditions and the size of the founder population or the availability of hosts, become critical for establishment.

This analysis was based on habitat suitability for the two principal dengue vector species, *Ae. aegypti* and *Ae. albopictus*. It was assumed that these two species do not interact, that is, the probability of the presence of each is independent of that of the presence of the other. The relative ecological risk for the establishment for each species was estimated using a global species distribution model at a 1 arc-minute resolution [15], [27] based on a maximum entropy algorithm incorporated in the Maxent software package [28]–[29]. Maxent was used because it has proven to be predictively superior to other species distribution modeling algorithm in a large variety of studies [27], [30].

As input, Maxent uses species occurrence points (presence-only data) and environmental layers (the explanatory variables). Distributional data were obtained from the Disease Vectors Database [31]. Coverage was global but not uniform with an overrepresentation of *Ae. aegypti* in Australia that may bias model results (data not shown). At the resolution of this analysis, there were 456 independent data points for *Ae. aegypti* and 117 for Ae. albopictus. Typically, about 30 points suffice for accurate SDM construction for mosquito species [17]. Moreoer, since these methods use only presence-only data (rather than presence-absence data), lack of uniform coverage is generally not considered problematic [16]–[19].

The environmental layers consist of four topographic variables (elevation, aspect, slope, compound topographic index) and a standard set of 19 climatic variables. Elevation was obtained from the United States Geological Survey’s Hydro–1K digital elevation model (DEM) data set [32]. Slope, aspect, and compound topographical index were derived from the DEM using the Spatial Analyst extension of ArcMap 10. Climatic data were obtained from the WorldClim database and consisted of 19 bioclmatic variables (annual mean temperature, mean diurnal range, isothermality, temperature seasonality, maximum temperature of warmest month, minimum temperature of coldest month, temperature annual range, mean temperature of wettest quarter, mean temperature of driest quarter, mean temperature of warmest quarter, mean temperature of coldest quarter, annual precipitation, precipitation of wettest month, precipitation of driest month, precipitation seasonality, precipitation of wettest quarter, precipitation of driest quarter, precipitation of warmest quarter, and precipitation of coldest quarter) [33]. Models were constructed using a variety of subsets of these environmental variables as described in Sarkar et al. [19]. All computations used default settings [30]. Averages over 100 replicate models were computed in Maxent.

Model performance was judged using the Akaike Information Criterion (AIC) for species distribution models as incorporated in the ENMTools software package [34]. Since the best model was signigicantly better than the others (*p*<0.05), no further criterion to break potential ties was needed. The best model for *Ae. aegypti* is one that used all 23 explanatory variables; that for *Ae. albopictus* is based on elevation, slope, aspect, maximum temperature of warmest month, minimum temperature of coldest month, precipitation of wettest month, and precipitation of driest month.

**Figure 1:**
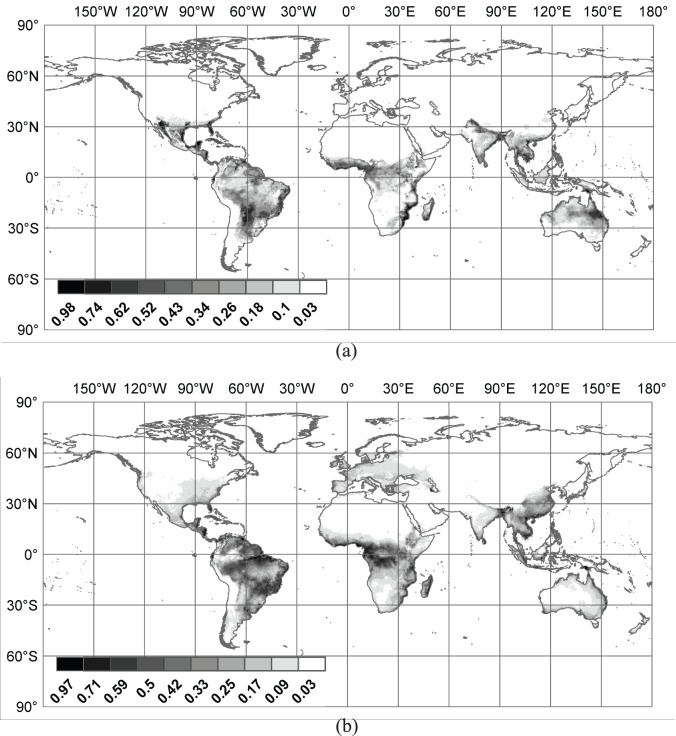
Map illustrating the Species Distribution Models for (a) *Ae. aegyptii* and (b) *Ae. Albopictus* [19]. The numbers on the scale are the predicted probabilities of presence.

The best models for *Ae. Aegypti* (Figure 1.a) and *Ae. Albopictus* (Figure 1.b) are reported as part of the results. The output from Maxent consists of relative suitability values between 0 and 1 which, when normalized, can be interpreted as the probabilistic expectation of vector presence of a species in a cell. The probabilistic expectation of at least one of the vector species being present in a cell was calculated as the complement of the probability that neither is present, assuming statistical independence. The expectations are aggregated to the city level by averaging them over all the cells in the relevant geographical units. These expectations define the relative ecological risk for dengue in each cell.

### 2.3 Mathematical Network Model

The proposed network model quantifies the relative risk of dengue infected travelers being present at any given airport. The risk posed by each airport is defined based on two criteria. Criteria I is *stopover risk*, defined as the total expected harm (or cost) posed by a given stopover airport *k* to regions other than airport *k*. The stopover risk is a function of the volume of passengers traveling through stopover airport *k,* and the origin-destination (OD) pairs they are traveling between. Criteria II is *destination risk*, defined as the total expected harm posed to destination airport *j* by all passengers arriving at airport *j*. The destination risk is a function of the volume of passengers traveling to destination airport *j,* and their route origins. Since, mathematically, both risks are expectations, which are additive, the sum of these two risks represents the total harm posed by a given airport.

Two previous mathematical models quantifying risk estimates for acquiring arboviral infection are by Massad and Wilder-Smith [35] and Codeco et. al. [36]. Massad and Wilder-Smith’s model was intended to evaluate the risk of infection at a specific site as a function of human population size, the number of infected mosquitoes, and estimated parameters for the biting rate and the probability that an infectious mosquito will infect a susceptible human. The model did not incorporate travel patterns or species distribution data; moreover, model predictions were not quantitatively validated using infection data. Codeco et. al. assessed the risk of yellow fever (YF) emergence in the city of Rio de Janeiro, Brazil, by estimating the probability of infected individuals arriving from YF-endemic areas via air and bus travel, and the probability of infective individuals triggering an epidemic (by using a stochastic transmission model). While this model accounted for travel patterns and local transmission probabilities, the model predictions were again not quantitatively validated.

#### 2.3.1 Problem formulation

**Figure 2:**
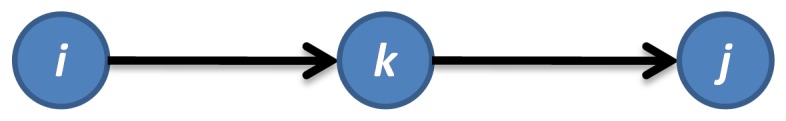
Schematic of a route with origin *i*, destination *j*, and stopover *k.*

In the proposed network structure, airports are represented as nodes, and the links in the network represent directed air travel connections between airports. Figure 2 illustrates an example travel route which originates at origin airport *i*, transfers through stopover airport *k*, and ends at destination airport *j*. There can be multiple stopover airports on a given travel route. In the data provided by IATA the maximum number of stops on any travel route is two. The notation used in the formal problem formulation is shown in Table 1.

**Table 1:** Problem Notation.

*i*:	Origin airport
*j*:	Destination airport
*k*:	Stopover airport
*t*:	Time period analyzed in the model
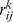 :	Total passenger volume on route *i-j-k*
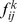 :	Total flow through stopover airport k, originating at airport *i* and ending at airport *j*
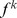 :	Total flow through stopover airport *k*
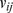 :	Total flow originating at airport *i* and ending at airport *j* (includes direct and stopover routes)
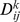 :	Distance of route *i-k-j*, that originates at *i,* ends at *j* goes through stopover k
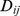 :	Distance of route *i–j*, that originates at *i* and ends at *j*
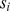 :	Suitability at node *i*
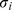 :	Outbreak intensity at node *i* (kept constant for now, outbreak size can be used as a proxy)
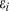 :	Endemic binary variable, equal to 1 if region is endemic, 0 o/w
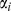 :	Population at *i*
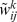 :	Harm posed to regions other than *k* by stopover airport *k* (function of flow along route *i-k-j)*
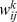 :	Relative harm posed to regions other than *k* by stopover airport *k* (function of flow along route *i-k-j)*
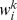 :	Relative harm posed to regions other than *k* by stopover airport *k* (function of flow originating at origin *i* which travels through *k*)
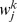 :	Relative harm posed to regions other than *k* by stopover airport *k* (function of flow ending at destination *j* which travels through *k*)
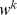 :	Total relative harm posed to regions other than *k* by stopover airport *k* (function of all flow traveling through *k)*
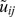 :	Harm posed to destination airport *j* by all flow originating at origin *i* and traveling to *j*
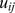 :	Relative harm posed to destination airport *j* by all flow originating at origin *i* and traveling to *j*
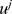 :	Total relative harm posed to destination airport *j* by all flow traveling to *j*
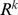 :	Total relative harm posed to airport *j* due to both stopover risk and destination risk

The total flow through each stopover airport *z* originating at *x* and ending at *y* is computed by aggregating the passenger volumes for all flights travelling from *x* to *y*, which stop at *z* at some point along the route for time period *t*. In this analysis the time period is equal to one month:
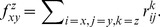
(1)


Given the total volume of flow along a route *i-k-j*, 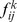, the total flow through each stopover airport *k* across all OD pairs can be computed:
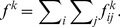
(2)


**Figure 3:**
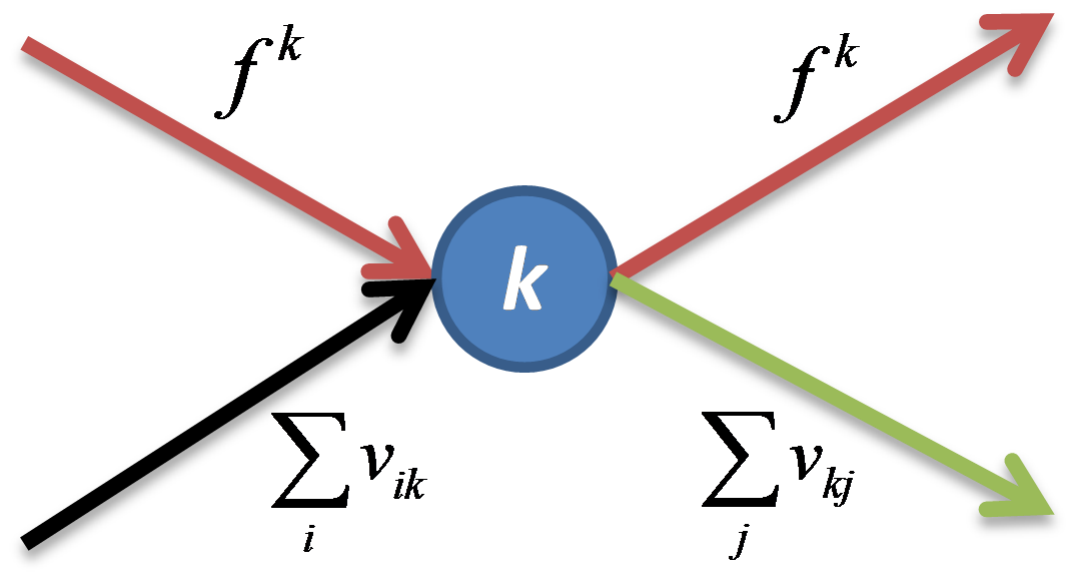
Schematic of all incoming and outgoing flow at node k.

The flow at any given airport is comprised of the stopover flow transferring at airport *k*, 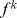, the total flow originating at airport *k*, 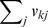, and the total flow ending at airport *k,* 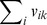. The flow at a given node is illustrated in Figure 3 below. There is no conservation of flow at node *k* because the total flow originating and ending at node *k* are independent. However all the stopover flow that enters node *k* must exit node *k*.

The harm posed by stopover airport *k* from travel originating at airport *i* and destined for airport *j* is defined by equation 3:
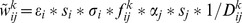
(3)


This model quantifies the risk transfer through an airport. The model is not intended to quantify risk of establishment at the stopover airport; rather that is quantified at the destination airport in the *destination risk* model presented in the following section. Equation (3) is specific to the OD pair (*i,j*), and is dependent on the origin being in a dengue-endemic region, the outbreak intensity at the origin, the suitability at the origin, the total passenger volume traveling through *k* between (*i,j*), the population at the destination, the suitability at the destination and the travel distance. The values computed using equation (3) are normalized by dividing by the highest value computed over all (*i,j,k*) combinations. The normalized risk, 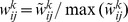, represents the *relative harm* posed by stopover airport *k* from travel originating at airport *i* and destined for airport *j*. The endemic binary variable, 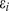, is included because only routes originating in dengue-endemic regions are assumed to have infected outgoing travelers. The origin suitability, *s_i_* is included to represent the relative ecological risk of dengue existing at the origin, and can serve as one proxy for the likelihood of an outgoing traveler being infected. The destination suitability, *s_j_*, is included because in order for a disease to continue to spread after being introduced into a new region by an infected traveler the destination habitat must be ecologically suitable for the spreading vector to exist and/or establish itself. The outbreak intensity, 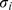, is a function of the outbreak size and population density at the origin, which can also serve as a proxy for an outgoing traveler being infected. The outbreak intensity variable is set to a constant in this work due to a lack of available data. The total passenger volume along route (*i,j*) through airport *k*, 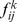, is captures the potential migration of the disease. Intuitively, the higher the passenger volume into airport *k*, the more likely an infected traveler will arrive at airport *k*, all other things being equal. The population at the destination, 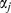, captures the threat posed to a given region if the disease was to be introduced. The destination airport parameters in the *stopover risk* model are necessary to capture the contribution of the final location of the passenger with the disease agent. As an example, consider two different travel routes: On Route 1 passengers travel from Miami to Winnipeg with a stopover in Houston; on Route 2 passengers travel from Miami to New Orleans with a stopover in Atlanta. In this case, airport surveillance of transfer passengers would be more beneficial in Atlanta to identify infected passengers traveling on to New Orleans where the spreading vector population exists, rather than in Houston, where infected travelers would be continuing on to Winnipeg where no local vector population exist and the habitat is ecologically unsuitable. Thus, where the infected passenger will end up represents the expected (negative) outcome (in analogy with utility theory), and is a necessary parameter for inclusion in the model in order to optimally allocate surveillance resources. Lastly, we assume that the risk is inversely proportional to the distance traveled which is correlated with the total time of transit. Our assumption was that longer travel time mitigates risk [14], [21] though the incubation and infection times for dengue can last much longer than travel times. Given that there was relatively little variation in distances between endemic areas and those with a high ecological suitability, this dependence was weak.

The harm posed by stopover airport *k* from travel route (*i,j*) is the same if it is the first or second stop along the route, as the risk is solely a function of the attributes of the origin and destination. Therefore the same risk function is applied to all stops along a route *i,j*.

Once the stopover risk, 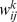, is computed as above, we can aggregate across origins, destinations, or both to identify the origins and destinations most susceptible and most liable. Specifically the following risks can be assessed:

(i) The expected relative harm posed by stopover airport *k* originating at airport *i* (equation 4),
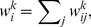
(4)


(ii) The expected relative harm posed by stopover airport *k* destined for airport *j,* (equation 5), and.
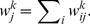
(5)


(iii) The total expected relative harm posed by stopover airport *k* (equation 6):
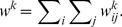
(6)


The harm posed to a destination airport *j* from travel originating at airport *i* is defined in equation 7. The set of variables and reasoning is similar to that in the stopover risk calculated previously. The main difference is the passenger flow variable, 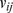, is now used in place of 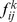. The total passenger volume originating at airport *i* and traveling to airport *j, v_ij_*, includes travel on both direct routes and indirect routes with stopovers. To quantify the destination risk we are only concerned with the total volume of passengers moving between regions in the time period considered; the actual route path does not impact the risk posed to a given destination:
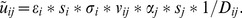
(7)


Similar to the stopover risk computed above, the values computed using equation (7) are normalized by dividing by the highest value computed over all (*i,j*) combinations. The normalized risk, 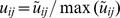, represents the *relative harm* posed to a destination airport *j* from travel originating at airport *i*. This relative harm can be aggregated across all origin airports to quantify the expected relative harm posed to destination airport *j,* as defined in equation 8:
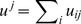
(8)


The previously computed stopover and destination risk can be summed to provide the expected relative harm contributed by each airport. This information can be used to prioritize airports where surveillance should be implemented. However the stopover and destination risk are also valuable independently, as they would require surveillance at different locations within the airport (i.e. stopover risk would require within airport surveillance, while destination risk requires surveillance upon exiting the airport). The total relative risk posed to airport *k* is illustrated in equation 9:
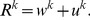
(9)


## Results

**Figure 4:**
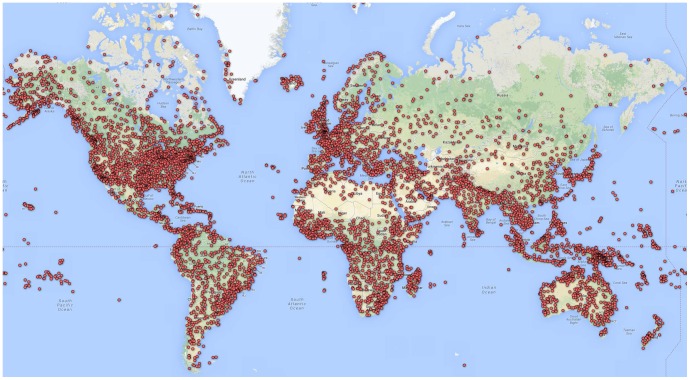
Map of all airports included in risk model.

Figure 4 illustrates the spatial distribution of all airports included in the model. Figures 1.a and 1.b depict the global Species Distribution Models (SDM) results for *Ae. Aegypti* and *Ae. Albopictus*, respectively. These results can be obtained by contacting either author.

The SDMs make some interesting preliminary predictions possible. The *Ae. albopictus* SDM designates suitable habitat along the west coast of the United States where persistent populations are yet to be reported; but specimens have been collected from two counties in Washington and California [37]. However, the SDM also predicts some suitable habitat at northern latitudes beyond the traditional northern limits [38]. The SDM predicts suitable habitat for *Ae. albopictus* in New Mexico; there is a report of possible establishment in that state [39]. It also predicts some suitable habitat for *Ae. albopictus* in Colorado; one report supports this prediction [40]. In Brazil, the SDM correctly predicts suitable habitat in the state of Ceará from which *Ae. albopictus* was collected for the first time in 2006 [41]. In Africa, *Ae. albopictus* was discovered in Bioko Island off Equatorial Guinea in 2003 [42], a highly suitable habitat according to the SDM. The Indian Ocean islands off the east coast of Africa, which are known to contain *Ae. albopictus*, are all predicted to have suitable habitat.

In Europe, since records from France and the Netherlands were used to construct the Maxent models, it is not surprising that the SDM predicts suitable habitat there. However, the SDM also identifies suitable habitat in Spain and Italy (including Sardinia), from which regions no data point was available, and published reports confirm those predictions [43]–[46]. In Australia, the SDM predicts suitable habitat in the northeast, in particular, in Cape York peninsula [47] as well as the Torres Strait Islands where infestation is widespread [48]. It finds large quantities of suitable habitat in northern New Zealand. Though the species has been intercepted there several times it does not yet appear to be established there [49]. The SDM correctly predicted suitable habitat for *Ae. albopictus* at the northern limits of its range in Hoshu island in Japan [50].

Turning to *Ae. aegypti*, it is instructive to compare the SDM to the 2005 range map for *Ae. aegypti* from the CDC [51]. The CDC map is based on political boundaries and therefore lacks fine resolution. Moreover, it is a map of *Ae. aegypti* occurrence and not suitable habitat. Compared to the SDM, it underestimates the range in the United States, Brazil, and Australia but overestimates the range in southern Africa. It also excludes the area around the Caspian Sea. For Asia and Australiasia, the maps are very similar with the SDM allowing suitable habitat to be identified at a much finer resolution. All countries that have had recognized DHF outbreaks [52] have suitable habitat for *Ae. aegypti* according to the SDM. For the U.S., the SDM predicts the existence of suitable habitat for *Ae. aegypti* in southern Arizona and New Mexico, that would usually have been presumed to be too dry for this species. However, anthropogenic modification of land cover, especially the creation of artificial water bodies, may have made the habitat more hospitable to *Ae. aegypti* which has already possibly established itself in some counties just north of the Mexican border [53]–[54].

**Figure 5:**
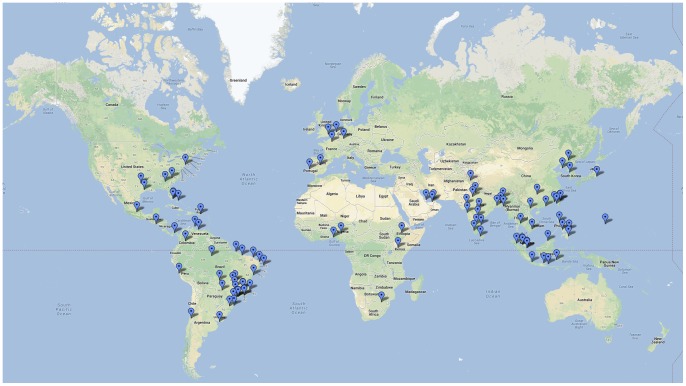
Map illustrating top 100 stopover risk airports identified by the model.

**Figure 6:**
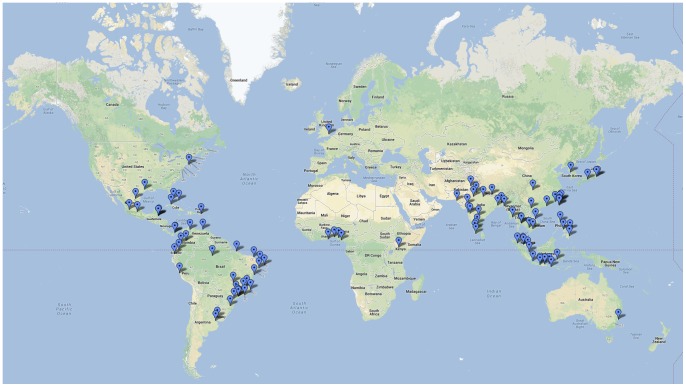
Map illustrating top 100 destination risk airports identified by the model.

The most important results of the analysis were the prioritization of stopover and destination airports on the basis of risk. The 100 highest risk stopover airports and destination airports are shown in Figures 5 and 6, respectively. Tables 2 and 3 list, respectively, the high risk destination and stopover airports which are located in non-endemic regions. Table 4 lists the high risks U.S. airports in non-endemic regions, and their corresponding regional suitability. Tables S1 and S2, included as supporting documents, contain the full list of the top 100 high risk airports, their rank and corresponding relative risk for both stopover risk and destination risk, respectively. These tables show that 14 and 26 of the destination and stopover airports in non-endemic regions, respectively, lie in the top 100 highest risk category. For stopover risk, six of the top 10 highest risk airports are in Brazil; no airport in India occurs in the top 10 in spite of its higher population total and population density–only two Indian airports (Delhi and Kolkata) occur in the top 20, in the latter case in spite of low passenger volume. Seven of the top 20 highest risk stopover airports are located in Southeast Asia. Destination risk is more evenly spread across the endemic regions; Brazil has three airports in the top 10 (three in the top 20) and India has two in the top 10 (four in the top 20), and five of the top 10 are in Southeast Asia. In addition, both the Changi Airport in Singapore and Hong Kong International Airport in Hong Kong are located in the top 20 highest risk for both stopover and destination airports; both these airports are among the top 20 busiest airports in the world. There are few African airports in either category; the Murtala Muhammed Airport in Nigeria is the only African airport that falls in the top 20 of either list, ranking 11^th^ in destination risk.

**Table 2:** Airports in List of Top 100 Destination Risk located in non-endemic regions.

IATA code	Airport City	Airport Name	Airport Country
AEP	Buenos Aires	Arpt. Jorge Newbery	Argentina
EZE	Buenos Aires	Ezeiza Ministro Pistarini	Argentina
SYD	Sydney	Kingsford Smith	Australia
CKG	Chongqing	Chongqing JiangbeiInternational	China
ACC	Accra	Kotoka	Ghana
NRT	Tokyo	Narita	Japan
HND	Tokyo	Haneda	Japan
KIX	Osaka	Kansai International	Japan
SIN	Singapore	Changi	Singapore
ICN	Seoul	Seoul (Incheon)	South Korea
LHR	London	Heathrow	United Kingdom
JFK	New York	John F Kennedy Intl	United States
MIA	Miami	Miami InternationalAirport	United States
IAH	Houston	George Bush Intercntl.	United States

**Table 3:** Airports in List of Top 100 Stopover Risk located in non-endemic regions.

IATA code	Airport City	Airport Name	Airport Country
BWN	Bandar Seri Begawan	Brunei International	Brunei Darussalam
SCL	Santiago	Arturo Merino Benitez	Chile
TEN	Tongren	Tongren	China
CNI	Changhai	Changhai	China
TNH	Tonghua	Tonghua Liuhe	China
CDG	Paris	Charles De Gaulle	France
FRA	Frankfurt	Frankfurt International Airport (Rhein-Main)	Germany
GUM	Guam	Guam International	Guam
NRT	Tokyo	Narita	Japan
AMS	Amsterdam	Amsterdam-Schiphol	Netherlands
CUR	Curacao	Hato International Airport	Netherlands Antilles
LIS	Lisbon	Lisboa - Portela	Portugal
DOH	Doha	Doha	Qatar
SIN	Singapore	Changi	Singapore
JNB	Johannesburg	Johannesburg International	South Africa
ICN	Seoul	Seoul (Incheon)	South Korea
MAD	Madrid	Barajas	Spain
LHR	London	Heathrow	United Kingdom
MIA	Miami	Miami International Airport	United States
ATL	Atlanta	Hartsfield-Jackson Atlanta Int	United States
IAH	Houston	George Bush Intercntl.	United States
DFW	Dallas	Dallas/Ft Worth Intl	United States
CLT	Charlotte	Douglas	United States
FLL	Fort Lauderdale	International	United States
JFK	New York	John F Kennedy Intl	United States
MVD	Montevideo	Carrasco International Airport	Uruguay

**Table 4:** High Risk US Airports in non-endemic regions.

IATA code	Airport City	Airport Name	Airport Country	Suitability
**U.S. Airports in Top 100 Stopover Risk in Non-Endemic Regions**				
MIA	Miami	Miami International Airport	United States	0.892
ATL	Atlanta	Hartsfield-Jackson Atlanta Int	United States	0.134
IAH	Houston	George Bush Intercntl.	United States	0.694
DFW	Dallas	Dallas/Ft Worth Intl	United States	0.096
CLT	Charlotte	Douglas	United States	0.085
FLL	Fort Lauderdale	International	United States	0.861
JFK	New York	John F Kennedy Intl	United States	0.290
**U.S. Airports in Top 100 Destination Risk in Non-Endemic Regions**				
JFK	New York	John F Kennedy Intl	United States	0.290
MIA	Miami	Miami International Airport	United States	0.892
IAH	Houston	George Bush Intercntl.	United States	0.694

Many of the high risk airports identified by the network-based risk model as likely to import dengue infected passengers also lie in regions predicted to be suitable habitats for the principle vector species by the SDM. For instance, in Europe, the risk model identified Charles De Gaulle Airport (CDG) in Paris, France, Amsterdam-Schiphol Airport (AMS) in Amsterdam, Netherlands and Barajas Airport (MAD) in Madrid, Spain as likely candidates for carrying infected stopover passengers. These three airports all lie in regions identified as suitable habitats by the SDM model. Travellers through these airports carry the potential for local transmission through the introduction of certain disease parasites into the local mosquitoes’ population. Such transmission has been documented resulting in “airport malaria” and attributed to individuals coming from malaria-endemic regions [55]–[60].

The risk model identified Narita Airport (NRT) in Tokyo, Japan as both a high risk stopover and high risk destination airport, while the SDM also identified Japan as a potentially habitable environment for the vector species. Two other airports in Japan, Haneda Airport (HND) in Tokyo and Kansai International Airport (KIX) in Osaka, were identified as high risk destinations as well. While the risk model reveals travellers to and through these airports to be likely candidates for carrying dengue, the results from the SDM imply the additional risk of an autochthonous transmission cycle resulting in the surrounding regions due to the suitable habitats.

Over the past decade travel acquired dengue cases have been increasingly reported in multiple non-endemic regions [3]. Table 2 and Table 3 list the high risk destination and stopover airports which are located in non-endemic regions, respectively. These tables include airports located in major cities such as Buenos Aires, Sydney, Tokyo, London, and New York City. The potential harm each of these airports pose, were an autochthonous transmission cycle to result, is further increased due to the large population size of the cities. Many airports listed in Table 3 represent major hubs for travel between tropical regions such as Southeast Asia and the western world. Multiple airports in the United States and Europe are among those likely to transfer infected passengers, as well as the Doha airport in Qatar, Johannesburg International Airport in South Africa, Changi Airport in Singapore, among others. In order to reduce the potential role of these airports in the further spread of dengue infected passenger’s into new regions targeted surveillance measures must be implemented.

Table 4 highlights both the stopover and destination airports in the United States which are at risk of importing infected passengers, and their corresponding suitability variable based on the SDM. Most significant are the airports in highly suitable environments, thus likely to import infected passengers who will remain in the region. Both Miami International Airport in Miami, Florida George Bush International Airport in Houston, Texas fit this criterion. In addition, the International Airport in Fort Lauderdale, Florida is also a highly suitable environment and likely stopover for dengue infected passengers.

Lastly, it is interesting to note that the airports identified by the risk model are not necessarily the busiest airports with respect to passenger volume (see Table S3). For instance, only 21 airports in the list of top 100 destination risk airports are among the top 100 busiest airports in terms of passenger travel volume. While 33 airports from the list of top 100 stop over risk airports are included in the top 100 busiest airports. This suggests it is important to consider other factors beyond travel volume when assessing airport risk.

## Discussion

Dengue currently presents a serious risk to many parts of the world where suitable environmental conditions for vector species’ occurrence and establishment provide the potential for local outbreaks, were the virus to be introduced. For dengue to be established in an autochthonous transmission cycle in a non-endemic region, the satisfaction of one critical criterion is likely necessary, and that of a second is likely to be important. The critical necessary criterion is that the area into which the infection is imported contains suitable habitat for the long–term persistence of dengue vectors. The likely criterion is that, at the time at which the infection arrives, local vector populations must be abundant or expanding in size for there to be potential disease outbreak/establishment.

The motivation for this analysis was the increasing number of dengue cases being reported, coinciding with an increase in both the prevalence of dengue worldwide and increased volume of international passenger air traffic originating from dengue-endemic regions since the 1990s. The aim of this paper was, therefore, to develop a preliminary integrated quantitative model that specifies the relative likelihood of the establishment of dengue in non-endemic areas based on passenger air travel patterns. More specifically, the model quantifies the relative risk posed to each stopover and destination airport based on the likelihood of a given air travel route carrying infected passengers. Besides passenger travel patterns, the model incorporates predictive species distribution models for the principal vector mosquito species.

However, before we discuss the practical implications of this model, the following limitations must be noted. First, with respect to the SDMs, as noted earlier, the mosquito distributional data were global but not uniform in coverage with an overrepresentation of *Ae. aegypti* in Australia. While lack of uniform coverage is generally not considered problematic in the type of analysis we use [16]–[19], further collection of data points, particularly from Africa, would add confidence in the results. Second, the ecological risk model assumed that there was no competition between *Ae. aegypti* and *Ae. albopictus*. Whether this is a reasonable assumption is simply not known: for us it served as the simplest assumption in the absence of data suggesting otherwise. The third limitation is due to a lack of available infection data. In the model the value of the outbreak intensity variable is set to one. This variable is intended to serve as a proxy for an outgoing traveler being infected, but requires time-dependent data on the outbreak size at a given origin. Infection reports for many countries were unavailable at this time of this study, even at the annual level, let alone the city and monthly level which would be consistent with the scale of the proposed model. Future improvements in surveillance and case reporting will allow for more precise quantification of this variable, further increasing the value of the model. Last, the set of dengue-endemic regions used to identify high risk origins was based on those identified by the CDC [26]. It is likely that the set of dengue-endemic regions is changing as the range of the disease has expanded during the last decade in which case the set of endemic regions specified in the model may require expansion and our entire analysis in need of reiteration.

The results from the model suggests there are major cities in the United States, Europe, Japan, and Australia, among others, all where dengue is not currently endemic, with a high risk of importing dengue infected travelers. Furthermore, many of the cities identified by the risk model, such as Miami, Florida and Houston, Texas in the United States are also at risk of dengue becoming established due to the highly suitable environmental conditions as predicted by the SDMs. The ability of the model to identify and rank airports based on the risk they pose can lead to more specific surveillance recommendations than, for instance, what the CDC is currently able to make with respect to prioritizing optimal airports for passenger surveillance efforts. In general, quantitative results from the model can aid in efficient and economic design of control measure strategies.

However, what this analysis mainly shows is that much of the high risk is driven by travel connections between airports in endemic regions. Most of the high stopover risk airports are in endemic regions of South America and Asia, especially in Brazil. For destination risk, there is a similar pattern but with India also figuring prominently. There are few African airports in either category, likely due to lower passenger volumes. This pattern suggest that, to arrest the continuing spread of dengue, more attention needs to be directed to airports in endemic region in comparison to those outside (in United States and the European Union).

While many control measures exist that can be effective in controlling the spread of insect-borne disease during air travel, it is impractical to implement all measures at all at risk locations. For agencies designing such control strategies, predictive risk models can be used to identify the most economical use of available surveillance resources. The general categories of control measures for Dengue Fever which are most common in practice are discussed below.

***Disinsection*** procedures involve the use of pesticides in aircrafts in order to exterminate insects, which are known vectors. Disinsection has been shown to be very efficient [61]–[62]. Although disinsection can effectively prevent importation of known vectors, the health issues associated with exposing passengers and crews to pesticides have been recorded, and must be further examined [63]–[65], As well as the importation potential from vectors surviving air travel in areas outside the main cabin [61].

A second option is containment strategy. The objective of ***containment*** strategies is to isolate infected people and minimize the number of infected individuals exposed to vectors that can further spread the disease, thereby decreasing the probability of subsequent infections. In order to prevent increased numbers of susceptible individuals to be exposed to known vectors, planning agencies may limit accessibility from or to endemic areas. In extreme cases, containment strategies may include the closing of airports. Additional containment measures may include travel restrictions on affected areas to contain the spread of the disease. Airline routes may be modified to minimize indirect multi-stop routes to destinations. Emergency measures are already implemented at times to deal with potential epidemics. To efficiently employ such containment strategies it is integral to have quantitative models such as that proposed in this paper which can rank airports and travel routes based on the potential risk they pose. The information can be used to reduce inter-region vector flows in emergency response situations by restricting air travel between certain high risks destination pairs. The same data can be used for proactive risk mitigation, when developing new flight schedules.

Thirdly, ***screening*** ** ***procedures*** can allow arrival and departure terminals to limit the frequency of successful importation into new areas due to air travel by known vectors. The Taiwan CDC has implemented a Rapid Dengue Blood Screening with the purpose of quarantining individuals who exhibit suspicious symptoms. The objective of the screen test is to prevent spreading of the disease due to native competent vectors during the viremia period. Screening of passengers presents complex challenges: Firstly, development of accurate screening tests is paramount, as unnecessarily quarantining individuals based on faulty results would not result in a feasible system. Secondly, the screening process must be fast enough to be accepted by passengers in specific locations. Similar to containment strategies, quantitative models which can identify high risk airports where screening should be implemented are required. Additionally, the proposed model is able to differentiate between risk from arriving and risk from stopover passengers, which require different screening policies.

A final option is vector control. ***Vector control*** is based on the idea of reducing transmission of the disease by targeting site-specific activities which are cost-effective. Vector control strategies range from large-scale implementation of bed nets to large-scale pesticide treatment in an effort to eradicate vectors. Other vector control strategies include larval control, and environmental control. Larval control can only be applied efficiently in areas where the mapping and characterization of breeding sites is well-documented. None the less vector control is of vital importance in control of global insect-borne diseases. Implementing sustainable and effective preventative and detection measures to contain epidemics in high-risk areas can be challenging and costly. It is, therefore, necessary to be selective in choosing the most appropriate set of intervention measures.

A model that accounts for potential control measures, in particular the costs (operational, monetary, organizational), and benefits (decreased importability and establishment of vectors, decreased probability of epidemics given establishment) of such control measures must be developed. While, ultimately, the primary target is to limit the number of people affected with such diseases, several other benefits can be identified:

- Decreasing the time necessary to identify disease outbreaks through the analysis of spatiotemporal data can allow for improved diagnosis capabilities in the health sector. Timely diagnosis is particularly important in areas where insect-borne diseases are not endemic, and as such, not expected.
- Identifying efficient mitigation strategies in a timely manner can save agencies significant resources by curbing the overall magnitude of disease episodes. The earlier outbreaks or epidemics can be identified and subsequently contained, the more efficient the use of resources will be.

The development of predictive risk models is an integral step in improving local and regional surveillance efforts. The explicit effect that airports have in the spread of diseases has yet to be fully quantified. However, the analysis reported here takes a significant step towards that end. Given that there is currently no vaccine for dengue, surveillance and intervention, along with vector control, remain the only relevant options to prevent further geographic spread of the disease. Results from the risk model and SDM together provide the means to evaluate and address the risk posed to currently non-endemic regions, and reduce the likelihood of dengue becoming established there. Meanwhile, the limitations of this analysis highlight the need for improving the quality of readily accessible disease data so as to enhance the prediction and control of epidemic episodes of vector-borne diseases in susceptible countries.

This model could be directly applied to Chikungunya and other diseases transmitted by Aedes albopictus [66]. Additionally, the techniques developed here can be used for the analysis of other diseases in which air transmission plays a role [17], [20], [21], [36]. The network analysis is applicable to all such diseases; however, the ecological suitability analysis is restricted to vector-borne diseases, but extendible to those which, unlike dengue, involve reservoir species along with vector species as part of the disease cycle [5], [16], [18], [19]. Finally the network model can potentially be extended in a multigraph framework to include other types of links–in the case of dengue, maritime transport links which have been implicated in the dispersal of Ae. albopictus [3].

## Supporting Information

Table S1 **Top 100 Stopover Risk Airports Ranked by Relative Risk.**
(PDF)

Table S2 **Top 100 Destination Risk Airports Ranked by Relative Risk.**
(PDF)

Table S3 **Top 100 Travelled Airports Ranked by Passenger Volume.**
(PDF)
