# Correction: A Global Airport-Based Risk Model for the Spread of Dengue Infection via the Air Transport Network

**DOI:** 10.1371/journal.pone.0194955

**Published:** 2018-03-21

**Authors:** Lauren Gardner, Sahotra Sarkar

There is an error in reference 4. The correct reference is: Gardner, L., Fajardo, D., Waller, S. T., Wang, O., and Sarkar, S. 2012. “A Predictive Spatial Model to Quantify the Risk of Air-Travel-Associated Dengue Importation into the United States and Europe.” Journal of Tropical Medicine 2012: Article ID 103679. DOI: https://doi.org/10.1155/2012/103679

The following information is missing from the Acknowledgments section: Figure 1 was generated by O. Wang using a template due to SS and V. Sanchez-Cordero. Thanks are due to them and to the following individuals for participating in the dengue project at the University of Texas: D. Fajardo, P. Illoldi-Rangel, M. Linaje, M. Pool, and S. Strutz.
