# Supplementary material for: A Global Airport-Based Risk Model for the Spread of Dengue Infection via the Air Transport Network
Source: PLoS One. 2013 Aug 29;8(8):e72129. doi: 10.1371/journal.pone.0072129 (PMC3756962; doi:10.1371/journal.pone.0072129)
Supplement: Table S1 — Top 100 Stopover Risk Airports Ranked by Relative Risk. (PDF) [file pone.0072129.s001.pdf]

Top 100 Stopover Risk Airports Ranked by Relative Risk

Rank	Relative Risk	IATA code	Airport city	Airport name	Airport Country
1	1.000	CGH	Sao Paulo	Congonhas	Brazil
2	0.819	BSB	Brasilia	Presidente Juscelino Kubitschek International	Brazil
3	0.577	GRU	Sao Paulo	Guarulhos Intl	Brazil
4	0.479	SIN	Singapore	Changi	Singapore
5	0.433	CGK	Jakarta	Soekarno-Hatta International	Indonesia
6	0.423	CNF	Belo Horizonte	Tancredo Neves International Airport	Brazil
7	0.413	VCP	Sao Paulo	Viracopos	Brazil
8	0.413	CWB	Curitiba	Afonso Pena International Airport	Brazil
9	0.357	BKK	Bangkok	International	Thailand
10	0.344	MNL	Manila	Ninoy Aquino Intl	Philippines
11	0.321	DEL	Delhi	Indira Gandhi Intl	India
12	0.316	HKG	Hong Kong	Hong Kong International	Hong Kong
13	0.311	GIG	Rio De Janeiro	Galeão–Antonio Carlos Jobim International	Brazil
14	0.275	BOG	Bogota	Eldorado	Colombia
15	0.268	KUL	Kuala Lumpur	Kuala Lumpur International Airport (klia)	Malaysia
16	0.260	SSA	Salvador	Arpt Luis R. Magalhaes	Brazil
17	0.260	CEB	Cebu	Mactan International	Philippines
18	0.203	MVD	Montevideo	Carrasco International Airport	Uruguay
19	0.202	CCU	Kolkata	Netaji Subhas Chandra	India
20	0.201	SDU	Rio De Janeiro	Santos Dumont	Brazil
21	0.179	BOM	Mumbai	Chhatrapati Shivaji	India
22	0.178	MEX	Mexico City	Juarez International	Mexico
23	0.168	HYD	Hyderabad	Begumpet Airport	India
24	0.160	PTY	Panama City	Tocumen International	Panama
25	0.159	SGN	Ho Chi Minh City	Tan Son Nhat	Vietnam
26	0.159	MAA	Chennai	Meenambakkam	India
27	0.157	POA	Porto Alegre	Salgado Filho	Brazil
28	0.156	BLR	Bangalore	Hal	India
29	0.153	REC	Recife	Guararapes Intl	Brazil
30	0.151	TPE	Taipei	Chiang Kai Shek	Taiwan
31	0.150	MIA	Miami	Miami International Airport	United States
32	0.137	DXB	Dubai	Dubai	United Arab Emirates
33	0.088	ABV	Abuja	International	Nigeria
34	0.082	VIX	Vitoria	Eurico Sales	Brazil
35	0.076	SUB	Surabaya	Juanda	Indonesia
36	0.072	FOR	Fortaleza	Pinto Martins	Brazil
37	0.070	ATL	Atlanta	Hartsfield-Jackson Atlanta Int	United States
38	0.060	MZG	Makung	Makung	Taiwan
39	0.059	TEN	Tongren	Tongren	China
40	0.058	GAU	Gawahati	Borjhar	India
41	0.057	BEL	Belem	Val De Cans	Brazil
42	0.057	LIM	Lima	J Chavez Intl	Peru
43	0.056	UPG	Ujung Pandang	Hasanudin	Indonesia
44	0.054	RAO	Ribeirao Preto	Leite Lopes	Brazil
45	0.054	DOH	Doha	Doha	Qatar
46	0.050	HAN	Hanoi	Noibai International	Vietnam
47	0.049	SAL	San Salvador	Comalapa International	El Salvador
48	0.048	DPS	Denpasar Bali	Ngurah Rai	Indonesia

49	0.044	IAH	Houston	George Bush Intercntl.	United States
50	0.042	DAC	Dhaka	Zia International	Bangladesh
51	0.041	AMD	Ahmedabad	Ahmedabad	India
52	0.040	FLN	Florianopolis	Hercilio Luz	Brazil
53	0.039	JNB	Johannesburg	Johannesburg International	South Africa
54	0.037	CCS	Caracas	Maiquetia	Venezuela
55	0.035	KNH	Kinmen	Shang-Yi	Taiwan
56	0.035	CGB	Cuiaba	M. Rondon	Brazil
57	0.034	ICN	Seoul	Seoul (Incheon)	South Korea
58	0.033	DFW	Dallas	Dallas/Ft Worth Intl	United States
59	0.032	CNI	Changhai	Changhai	China
60	0.032	UDI	Uberlandia	Eduardo Gomes	Brazil
61	0.031	GYN	Goiania	Santa Genoveva	Brazil
62	0.030	CLT	Charlotte	Douglas	United States
63	0.029	FLL	Fort Lauderdale	International	United States
64	0.029	SJU	San Juan	Luis Munoz Marin Intl	Puerto Rico
65	0.029	LOS	Lagos	Murtala Muhammed	Nigeria
66	0.028	NBO	Nairobi	Jomo Kenyatta International	Kenya
67	0.028	CDG	Paris	Charles De Gaulle	France
68	0.027	SLZ	Sao Luiz	Mal. Cunha Machado	Brazil
69	0.026	ADD	Addis Ababa	Bole	Ethiopia
70	0.026	NAT	Natal	Augusto Severo	Brazil
71	0.026	CGR	Campo Grande	Internacional	Brazil
72	0.024	MAD	Madrid	Barajas	Spain
73	0.022	DVO	Davao	Francisco Bangoy International Airport	Philippines
74	0.021	CMB	Colombo	Bandaranayake	Sri Lanka
75	0.020	TNH	Tonghua	Tonghua Liuhe	China
76	0.019	BWN	Bandar Seri Begawan	Brunei International	Brunei Darussalam
77	0.018	SJO	San Jose	Juan Santamaria Intl	Costa Rica
78	0.017	MAO	Manaus	Eduardo Gomes Intl	Brazil
79	0.016	AMS	Amsterdam	Amsterdam-Schiphol	Netherlands
80	0.015	PLU	Belo Horizonte	Pampulha	Brazil
81	0.015	BTH	Batam	Hang Nadim	Indonesia
82	0.015	LIS	Lisbon	Lisboa - Portela	Portugal
83	0.015	FRA	Frankfurt	Frankfurt International Airport (Rhein-Main)	Germany
84	0.014	GUM	Guam	Guam International	Guam
85	0.014	SXR	Srinagar	Srinagar	India
86	0.014	JFK	New York	John F Kennedy Intl	United States
87	0.014	TSA	Taipei	Sung Shan	Taiwan
88	0.014	COK	Kochi	Kochi	India
89	0.014	MES	Medan	Polania	Indonesia
90	0.014	CGP	Chittagong	Patenga	Bangladesh
91	0.013	NAG	Nagpur	Sonegaon	India
92	0.013	AUH	Abu Dhabi	Abu Dhabi Intl	United Arab Emirates
93	0.012	JAI	Jaipur	Sanganeer	India
94	0.012	SCL	Santiago	Arturo Merino Benitez	Chile
95	0.012	NAS	Nassau	Intl	Bahamas
96	0.012	NRT	Tokyo	Narita	Japan
97	0.012	CUR	Curacao	Hato International Airport	Netherlands Antilles
98	0.011	LHR	London	Heathrow	United Kingdom

99	0.011	SZB	Kuala Lumpur	Sultan Abdul Aziz Shah	Malaysia
100	0.010	ILO	Iloilo	Mandurriao	Philippines
