# Supplementary material for: A Global Airport-Based Risk Model for the Spread of Dengue Infection via the Air Transport Network
Source: PLoS One. 2013 Aug 29;8(8):e72129. doi: 10.1371/journal.pone.0072129 (PMC3756962; doi:10.1371/journal.pone.0072129)
Supplement: Table S2 — Top 100 Destination Risk Airports Ranked by Relative Risk. (PDF) [file pone.0072129.s002.pdf]

## Top 100 Destination Risk Airports Ranked by Relative Risk

| Rank | Relative Risk | IATA code | Airport city | Airport name | Airport Country |
|---|---|---|---|---|---|
| 1 | 1.000 | MNL | Manila | Ninoy Aquino Intl | Philippines |
| 2 | 0.637 | CGK | Jakarta | Soekarno-Hatta International | Indonesia |
| 3 | 0.579 | CGH | Sao Paulo | Congonhas | Brazil |
| 4 | 0.449 | BOM | Mumbai | Chhatrapati Shivaji | India |
| 5 | 0.313 | SDU | Rio De Janeiro | Santos Dumont | Brazil |
| 6 | 0.284 | DEL | Delhi | Indira Gandhi Intl | India |
| 7 | 0.257 | GRU | Sao Paulo | Guarulhos Intl | Brazil |
| 8 | 0.254 | BKK | Bangkok | International | Thailand |
| 9 | 0.206 | SGN | Ho Chi Minh City | Tan Son Nhat | Vietnam |
| 10 | 0.202 | HKG | Hong Kong | Hong Kong International | Hong Kong |
| 11 | 0.178 | LOS | Lagos | Murtala Muhammed | Nigeria |
| 12 | 0.113 | CCU | Kolkata | Netaji Subhas Chandra | India |
| 13 | 0.109 | SIN | Singapore | Changi | Singapore |
| 14 | 0.094 | DAC | Dhaka | Zia International | Bangladesh |
| 15 | 0.084 | NRT | Tokyo | Narita | Japan |
| 16 | 0.083 | SUB | Surabaya | Juanda | Indonesia |
| 17 | 0.072 | TZA | Belize City | Municipal | Belize |
| 18 | 0.068 | CNF | Belo Horizonte | Tancredo Neves International Airport | Brazil |
| 19 | 0.062 | BZE | Belize City | Philip S.W.Goldson Int | Belize |
| 20 | 0.062 | BLR | Bangalore | Hal | India |
| 21 | 0.058 | KUL | Kuala Lumpur | Kuala Lumpur International Airport (klia) | Malaysia |
| 22 | 0.057 | DMK | Bangkok | Don Muang Airport | Thialand |
| 23 | 0.057 | ICN | Seoul | Seoul (Incheon) | South Korea |
| 24 | 0.055 | MEX | Mexico City | Juarez International | Mexico |
| 25 | 0.052 | TSA | Taipei | Sung Shan | Taiwan |
| 26 | 0.052 | AEP | Buenos Aires | Arpt. Jorge Newbery | Argentina |
| 27 | 0.043 | HAN | Hanoi | Noibai International | Vietnam |
| 28 | 0.043 | HND | Tokyo | Haneda | Japan |
| 29 | 0.043 | KHH | Kaohsiung | International | Taiwan |
| 30 | 0.041 | TPE | Taipei | Chiang Kai Shek | Taiwan |
| 31 | 0.035 | CCS | Caracas | Maiquetia | Venezuela |
| 32 | 0.033 | REC | Recife | Guararapes Intl | Brazil |
| 33 | 0.030 | CGP | Chittagong | Patenga | Bangladesh |
| 34 | 0.029 | EZE | Buenos Aires | Ezeiza Ministro Pistarini | Argentina |
| 35 | 0.027 | FOR | Fortaleza | Pinto Martins | Brazil |
| 36 | 0.027 | SSA | Salvador | Arpt Luis R. Magalhaes | Brazil |
| 37 | 0.024 | CEB | Cebu | Mactan International | Philippines |
| 38 | 0.022 | UPG | Ujung Pandang | Hasanudin | Indonesia |
| 39 | 0.022 | HYD | Hyderabad | Begumpet Airport | India |
| 40 | 0.020 | MES | Medan | Polania | Indonesia |
| 41 | 0.020 | CWB | Curitiba | Afonso Pena International Airport | Brazil |
| 42 | 0.020 | JFK | New York | John F Kennedy Intl | United States |
| 43 | 0.019 | KIX | Osaka | Kansai International | Japan |
| 44 | 0.018 | KHI | Karachi | Quaid-E-Azam Intl | Pakistan |
| 45 | 0.017 | RGN | Yangon | Mingaladon | Myanmar |
| 46 | 0.017 | POA | Porto Alegre | Salgado Filho | Brazil |
| 47 | 0.016 | VCP | Sao Paulo | Viracopos | Brazil |
| 48 | 0.015 | BOG | Bogota | Eldorado | Colombia |

| | | | | | |
|---|---|---|---|---|---|
| 49 | 0.014 | NBO | Nairobi | Jomo Kenyatta International | Kenya |
| 50 | 0.013 | PLU | Belo Horizonte | Pampulha | Brazil |
| 51 | 0.013 | DPS | Denpasar Bali | Ngurah Rai | Indonesia |
| 52 | 0.012 | HAV | Havana | Jose Marti Intl | Cuba |
| 53 | 0.011 | PLM | Palembang | Mahmud Badaruddin Ii | Indonesia |
| 54 | 0.011 | BEL | Belem | Val De Cans | Brazil |
| 55 | 0.011 | SRG | Semarang | Achmad Yani | Indonesia |
| 56 | 0.011 | DVO | Davao | Francisco Bangoy International Airport | Philippines |
| 57 | 0.011 | PHC | Port Harcourt | Port Harcourt | Nigeria |
| 58 | 0.010 | KNH | Kinmen | Shang-Yi | Taiwan |
| 59 | 0.010 | PAC | Panama City | Paitilla | Panama |
| 60 | 0.009 | MAO | Manaus | Eduardo Gomes Intl | Brazil |
| 61 | 0.009 | GYE | Guayaquil | Simon Bolivar | Ecuador |
| 62 | 0.009 | COK | Kochi | Kochi | India |
| 63 | 0.009 | GYN | Goiania | Santa Genoveva | Brazil |
| 64 | 0.009 | LHE | Lahore | Alama Iqbal International | Pakistan |
| 65 | 0.009 | DAD | Da Nang | Da Nang | Vietnam |
| 66 | 0.008 | JOG | Yogyakarta | Adisutjipto | Indonesia |
| 67 | 0.008 | PNH | Phnom Penh | Phnom Penh International | Cambodia |
| 68 | 0.007 | MIA | Miami | Miami International Airport | United States |
| 69 | 0.007 | UIO | Quito | Mariscal Sucr | Ecuador |
| 70 | 0.007 | ACC | Accra | Kotoka | Ghana |
| 71 | 0.007 | RMQ | Taichung | ChingChuanKang | Taiwan |
| 72 | 0.006 | CKG | Chongqing | Chongqing Jiangbei International | China |
| 73 | 0.006 | NAS | Nassau | Intl | Bahamas |
| 74 | 0.006 | ILO | Iloilo | Mandurriao | Philippines |
| 75 | 0.006 | PTY | Panama City | Tocumen International | Panama |
| 76 | 0.006 | LKO | Lucknow | Amausi | India |
| 77 | 0.006 | TRV | Thiruvananthapuram | International | India |
| 78 | 0.006 | TNN | Tainan | Tainan | Taiwan |
| 79 | 0.005 | SZB | Kuala Lumpur | Sultan Abdul Aziz Shah | Malaysia |
| 80 | 0.005 | JAI | Jaipur | Sanganeer | India |
| 81 | 0.005 | NAT | Natal | Augusto Severo | Brazil |
| 82 | 0.005 | AMD | Ahmedabad | Ahmedabad | India |
| 83 | 0.005 | SJU | San Juan | Luis Munoz Marin Intl | Puerto Rico |
| 84 | 0.005 | IAH | Houston | George Bush Intercntl. | United States |
| 85 | 0.005 | MAR | Maracaibo | La Chinita | Venezuela |
| 86 | 0.005 | MTY | Monterrey | Gen Mariano Escobedo | Mexico |
| 87 | 0.005 | RAO | Ribeirao Preto | Leite Lopes | Brazil |
| 88 | 0.005 | VIX | Vitoria | Eurico Sales | Brazil |
| 89 | 0.005 | PNQ | Pune | Lohegaon | India |
| 90 | 0.005 | CLO | Cali | Alfonso B. Aragon | Colombia |
| 91 | 0.004 | AMI | Mataram | Selaparang | Indonesia |
| 92 | 0.004 | BNI | Benin City | Benin City | Nigeria |
| 93 | 0.004 | SYD | Sydney | Kingsford Smith | Australia |
| 94 | 0.004 | MCZ | Maceio | Palmares | Brazil |
| 95 | 0.004 | LHR | London | Heathrow | United Kingdom |
| 96 | 0.004 | LIM | Lima | J Chavez Intl | Peru |
| 97 | 0.004 | KTM | Kathmandu | Tribhuvan | Nepal |
| 98 | 0.004 | CUK | Caye Caulker | Caye Caulker | Belize |

| | | | | | |
|---|---|---|---|---|---|
| 99 | 0.004 | CJB | Coimbatore | Peelamedu | India |
| 100 | 0.004 | GDL | Guadalajara | Miguel Hidal | Mexico |
