# Supplementary material for: A Global Airport-Based Risk Model for the Spread of Dengue Infection via the Air Transport Network
Source: PLoS One. 2013 Aug 29;8(8):e72129. doi: 10.1371/journal.pone.0072129 (PMC3756962; doi:10.1371/journal.pone.0072129)
Supplement: Table S3 — Top 100 Travelled Airports Ranked by Passenger Volume. (PDF) [file pone.0072129.s003.pdf]

Top 100 Travelled Airports Ranked by Passenger Volume

Rank	IATA code	Airport City	Airport Name	Airport Country
1	ATL	Atlanta	Hartsfield-Jackson Atlanta Int	United States
2	PEK	Beijing	Capital	China
3	LHR	London	Heathrow	United Kingdom
4	ORD	Chicago	Ohare International	United States
5	HND	Tokyo	Haneda	Japan
6	LAX	Los Angeles	Los Angeles International	United States
7	CDG	Paris	Charles De Gaulle	France
8	DFW	Dallas	Dallas/Ft Worth Intl	United States
9	FRA	Frankfurt	Frankfurt International Airport (Rhein-Main)	Germany
10	HKG	Hong Kong	Hong Kong International	Hong Kong
11	DEN	Denver	Denver International	United States
12	DXB	Dubai	Dubai	United Arab Emirates
13	CGK	Jakarta	Soekarno-Hatta International	Indonesia
14	AMS	Amsterdam	Amsterdam-Schiphol	Netherlands
15	MAD	Madrid	Barajas	Spain
16	BKK	Bangkok	International	Thailand
17	JFK	New York	John F Kennedy Intl	United States
18	SIN	Singapore	Changi	Singapore
19	CAN	Guangzhou	Baiyun	China
20	LAS	Las Vegas	Mccarran Intl	United States
21	PVG	Shanghai	Pu Dong	China
22	SFO	San Francisco	San Francisco International	United States
23	PHX	Phoenix	Sky Harbor Intl	United States
24	IAH	Houston	George Bush Intercntl.	United States
25	CLT	Charlotte	Douglas	United States
26	MIA	Miami	Miami International Airport	United States
27	MUC	Munich	Franz Josef Strauss	Germany
28	KUL	Kuala Lumpur	Kuala Lumpur International Airport (klia)	Malaysia
29	FCO	Rome	Leonardo Da Vinci (Fiumicino)	Italy
30	IST	Istanbul	Ataturk	Turkey
31	SYD	Sydney	Kingsford Smith	Australia
32	MCO	Orlando	International	United States
33	ICN	Seoul	Seoul (Incheon)	South Korea
34	DEL	Delhi	Indira Gandhi Intl	India
35	BCN	Barcelona	El Prat De Llobregat	Spain
36	LGW	London	Gatwick	United Kingdom
37	EWR	Newark	Newark International Airport	United States
38	YYZ	Toronto	Toronto/Lester B. Pearson International	Canada
39	SHA	Shanghai	Hongqiao	China
40	MSP	Minneapolis	St Paul Intl	United States
41	SEA	Seattle	Seattle/tacoma International	United States
42	DTW	Detroit	Wayne County	United States
43	PHL	Philadelphia	Philadelphia International	United States
44	BOM	Mumbai	Chhatrapati Shivaji	India
45	GRU	Sao Paulo	Guarulhos Intl	Brazil
46	MNL	Manila	Ninoy Aquino Intl	Philippines

47	CTU	Chengdu	Chengdu	China
48	BOS	Boston	Logan International	United States
49	SZX	Shenzhen	Shenzhen	China
50	MEL	Melbourne	Tullamarine	Australia
51	NRT	Tokyo	Narita	Japan
52	ORY	Paris	Orly	France
53	MEX	Mexico City	Juarez International	Mexico
54	DME	Moscow	Domodedovo	Russia
55	AYT	Antalya	Antalya	Turkey
56	TPE	Taipei	Chiang Kai Shek	Taiwan
57	ZRH	Zurich	Zürich	Switzerland
58	LGA	New York	La Guardia	United States
59	FLL	Fort Lauderdale	International	United States
60	IAD	Washington	Washington Dulles Intl	United States
61	PMI	Palma Mallorca	Palma Mallorca	Spain
62	CPH	Copenhagen	Copenhagen Airport	Denmark
63	SVO	Moscow	Sheremetyevo	Russia
64	BWI	Baltimore	Balt/wash International	United States
65	KMG	Kunming	Kunming	China
66	VIE	Vienna	Schwechat International	Austria
67	OSL	Oslo	Oslo Airport, Gardermoen	Norway
68	JED	Jeddah	King Abdulaziz International	Saudi Arabia
69	BNE	Brisbane	Brisbane International	Australia
70	SLC	Salt Lake City	International	United States
71	DUS	Dusseldorf	Düsseldorf International Airport (Rhein-Ruhr)	Germany
72	BOG	Bogota	Eldorado	Colombia
73	MXP	Milan	Malpensa	Italy
74	JNB	Johannesburg	Johannesburg International	South Africa
75	ARN	Stockholm	Arlanda	Sweden
76	MAN	Manchester	Manchester	United Kingdom
77	MDW	Chicago	Midway	United States
78	DCA	Washington	Ronald Reagan National	United States
79	BRU	Brussels	National	Belgium
80	DUB	Dublin	Dublin	Ireland
81	GMP	Seoul	Gimpo International	South Korea
82	DOH	Doha	Doha	Qatar
83	STN	London	Stansted	United Kingdom
84	HGH	Hangzhou	Hangzhou	China
85	CJU	Jeju	Jeju Airport	South Korea
86	YVR	Vancouver	Vancouver Intl	Canada
87	TXL	Berlin	Tegel	Germany
88	SAN	San Diego	Lindberg Fld S. Diego	United States
89	TPA	Tampa	Tampa International	United States
90	CGH	Sao Paulo	Congonhas	Brazil
91	BSB	Brasilia	Presidente Juscelino Kubitschek International Airport	Brazil
92	CTS	Sapporo	Chitose	Japan
93	XMN	Xiamen	Xiamen	China
94	RUH	Riyadh	King Khaled Intl	Saudi Arabia

95	FUK	Fukuoka	Fukuoka	Japan
96	GIG	Rio De Janeiro	Galeão–Antonio Carlos Jobim International Airport	Brazil
97	HEL	Helsinki	Helsinki-vantaa	Finland
98	LIS	Lisbon	Lisboa - Portela	Portugal
99	ATH	Athens	Eleftherios Venizelos	Greece
100	AKL	Auckland	Auckland International	New Zealand
